# The Effectiveness of Group Training of CBT-Based Stress Management on Anxiety, Psychological Hardiness and General Self-Efficacy among University Students

**DOI:** 10.5539/gjhs.v8n6p47

**Published:** 2015-09-28

**Authors:** Hamdam Molla Jafar, Seddigheh Salabifard, Seyedeh Maryam Mousavi, Zahra Sobhani

**Affiliations:** 1 Health Psychology, Islamic Azad University, Karaj, Iran; 2 General Psychology, Iran University of Medical Sciences, Tehran, Iran; 3 Department of psychology, Rasht Branch, Islamic Azad Univrsity, Rasht, Iran

**Keywords:** stress management training, anxiety, psychological hardiness, general self-efficacy

## Abstract

**Background:**

Admission to university is a very sensitive period of life for efficient, active, and young workforces in any country, and it is mostly associated with many changes in social and human relationships. These changes lead to anxiety in students. Moreover, humans need certain functions in order to adaptively deal with different life situations and challenges. By training stress management, these functions can help human acquire the required abilities.

**Objective:**

The present study was aimed at investigating the effectiveness of stress management training in anxiety, psychological hardiness, and general self-efficacy among university students.

**Method:**

The study was a quasi-experimental intervention (pretest-posttest-follow-up) including a control group, it was a fundamental applied study. The statistical population consisted of all students of Islamic Azad University, Karaj, Iran. Convenient sampling was employed to select 30 students who were divided into an experimental group (n=15) and a control group (n=15). Before stress management training, both groups filled out Beck Anxiety Inventory, Long and Goulet scale of psychological hardiness, and General Self-efficacy Scale (GSE-10). Afterwards, the experimental group was provided with stress management training. And after the experiment, the abovementioned questionnaires and scales were responded by the two groups. Finally the collected data were analyzed and compared using one-way MANOVA.

**Results:**

The results of MANOVA indicated that there was a significant difference between the two groups in terms of anxiety, hardiness, and general self-efficacy (p<0.001).

**Conclusion:**

According to the results of the present study and those of previous investigations that are in agreement with those of the present study, it can be concluded that stress management among university students cause anxiety to drop; moreover, it enhances their psychological hardiness and self-efficacy. In regard with the role and importance of stress management, training this skill should be included in educational plans of university.

## 1. Introduction

Anxiety is a physiological condition that includes cognitive, physical, emotional, and behavioral components, and everybody experiences it in their lives (Isra et al., 2008). Presence of an acceptable level of anxiety is considered as an adapted response which brings about humans’ different position taking against natural and unnatural events. Therefore, presence of a little anxiety can have a positive effect on the process of life and its revolution. It is natural that this type of anxiety not only causes no damage to human’s growth process but also it is considered as a useful event (Hatami & Ardalan, 2010). However, a high level of anxiety can cause different types of diseases.

The results of different studies indicated that there has been an increase in prevalence and intensity of remarkable problems of mental health among university students (Zivin et al., 2009). Depression and anxiety are the most common psychological problems among university students (Erol Deirbatir, 2012; Raja et al., 2012). Some have reported prevalence of psychological disorders to be 12.75-30.4% among Iranian university students (Saheb Al-zamani, 2010). University students’ mental health is one of the essential affairs of student life, and paying attention to it can have a direct effect on the growth and development of the community and is the main factor in optimal exploitation of efficient educated workforces (Shirbim et al., 2008).

Timmerman et al. (1998) studied the effects of stress management lesson planning. The results of their study indicated that this type of training can reduce mental pressure, anxiety, and daily involvements and increase courage and satisfaction. Halamandaris and Power (1999) concluded that training behavioral or muscular relaxation could reduce the students’ anxiety. Mizuno et al. (1999) stated that there is a significant relationship between mental health and problem-oriented coping style, and that training how to deal and control stress significantly reduces the individuals’ scores on anxiety, depression, and social dysfunction. The results of the study conducted by Shirbim, Sudani, and Shafi’ Abadi (2008) indicated that training stress management enhances mental health and reduces physical symptoms, anxiety, social dysfunction, and depression. Therefore, one of the objectives of the present study is to examine the effectiveness of stress management training on anxiety among university students.

Moreover, another variable that was considered in the present study is psychological hardiness. With an increasing growth of awareness in the field of psychology and advent of new scopes, the concept of hardiness has been paid unique attention by positivist psychologists. In this framework, hardiness is defines as a combination of attitudes and beliefs that give an individual motivation and courage so that he can perform well and strategically in the face of pressure-causing and difficult situations and try to adapt himself; therefore, he can step toward his growth and development through unfavorable and catastrophic events (Maddi, 2002).

Kobasa, Maddi, and Zola (1983) defined hardiness as a combination of beliefs about oneself and the universe that is composed of components like commitment, control, and competitiveness. Belief in change, revolution, dynamism of life, and the attitude that there is no event as a threat to human security and health can cause cognitive flexibility and tolerance against stress-causing difficult events and vague situation. Maddi, Wadha, and Haier (1996) and Kobasa and Puccetti (1983) believe that psychological features of hardiness such as remarkable curiosity, tendency to have interesting and meaningful experiences, self-expression, liveliness, and the belief that change in life is natural can help with the individuals’ adaptation to the life stressful events. Investigations indicate that there is a positive relationship between physical health and mental health, and as an internal tolerance resource, it reduces negative effects of stress and prevents the appearance of mental and physical discrapancies (Kobasa, 1979; Florian, Mikulincer & Yaubman, 1995; Brooks, 2003). Therefore, the second objective of the present study is to investigate the effectiveness of stress management training on psychological hardiness.

Moreover, self-efficacy is another variable that was investigated in the present study. Self-efficacy is a key construction in social cognitive theory (Bandura, 2004). Social cognitive theory is derived from an agentic perspective in which the individuals’ behavior is perceived in a purposeful and predictable way, and they regulate their motivation and behavior through active self-assessment. According to this perspective, individuals with beliefs of self-efficacy can control their feelings and behavior using their beliefs through a working model, and the individuals’ behavior is predictable through these beliefs (Bandura, 2004).

Bandura (2004) believes that self-efficacy or the individual’s judgment about his abilities and capacities to control his life events has the greatest impact on his actions and self-regulation. It assesses the important choices of the individual’s life procedure. Moreover, devotion of necessary attempt to actualization of activities, endurance against problems, and unsuccessful experiences of life are affected by self-efficacy.

One of the reasons for significance of studying general self-efficacy among university students is that, on the one hand, there is a relationship between this variable and mental disorder. In this regard, research studies have concluded that mental disorders occur when there is a low level of self-efficacy. The results of one of the studies that were conducted by Rezayat and Dehghan Nayeri (2013) indicated that there is a significant relationship between high depression and low self-efficacy. Moreover, Bakhtiyarpour, Hafezi, and Shini (2010) concluded that exam anxiety can be predicted through self-efficacy. On the other hand, another reason for significance of studying general self-efficacy among students is that there is a relationship between self-efficacy and the students’ academic achievement, which can be understood from the results of the study conducted by Abolghasemi and Javanmiri (2012). The results of their study indicated that academic achievement can increase as a result of a high level of self-efficacy. Moreover, Tamadoni, Hatemi, and Zarrini (2011) concluded that there is a significant relationship between self-efficacy and academic achievement. Therefore, due to the high significance of self-efficacy among university students, measures that are adopted to enhance this variable are highly significant. Stress management lesson planning is one of these measures. The results of the study conducted by Hardin et al. (2002) indicated that psychological measures cause the individuals’ understanding of stress, self-efficacy, and social support to rise. The results of the study conducted by Abolghasemi et al. (2012) training life skills can affect the spouses’ self-efficacy of the addicts. Since stress management is one of the life skills, along with anxiety and psychological hardiness, the present study investigates the effectiveness of stress management training on self-efficacy among university students.

## 2. Method

The study was a quasi-experimental intervention (pretest-posttest-follow-up) including a control group, it was a fundamental applied study.

### 2.1 Statistical Population and the Study Sample

The statistical population included all students of Islamic Azad University, Karaj Iran. Convenient sampling was utilized to select 30 students as the study sample that was assigned into an experimental group and a control one. In the present study, stress management lesson plan was the independent variable, and anxiety, psychological hardiness, and self-efficacy were dependent variables. Beck Anxiety Inventory, Long and Goulet scale of psychological hardiness, and General Self-efficacy Scale (GSE-10) were administered before and 45 days after the investigation.

Mean and standard deviation were employed to describe the collected data. Variance analysis was employed to compare the collected data. And statistical analyses were carried out through SPSS 16.0.

### 2.2 Instrumentation

A. Beck Anxiety Inventory (BAI): Beck Anxiety Inventory is a self-report questionnaire that is composed of 4-option 21 questions that are scored in the range of 0-3. The total score ranges from 0 to 63. The results of the conducted studies in Iran indicate that this questionnaire has a high level of reliability and validity. For example, Ghorayi (1993) reported its reliability to be 0.80. Moreover, Kaviyani and Mousavi (1999) reported its reliability and Cronbach’s alpha to be 0.72 and 0.92, respectively.

B. Long and Goult Hardiness Scale (LGHS): It is a self-report questionnaire that is composed of 45 questions. It developed by Long and Goult (2003) based on the definition of psychological hardiness construction in order to assess this variable in certain stressful situations. This scale contains 3 subscales of control subscale (16 question), commitment (16 questions), and challenge seeking (16 question). The questionnaire’s items are scored based on a 5-point Likert scale where 1 indicates “completely disagree” and 5 stands for “completely agree”. The respondent’s total score is calculated from the sum of the scores obtained from each question of the questionnaire. In the study conducted by Long and Goult (2003), the results of the reliability of the scale indicated that the correlation between the total scores of the questionnaire and the respondents’ scores in the subscales was average and high, and the Cronbach’s alpha indicated a good level of internal consistency of the scale (Long & Goult, 2003).

C. General Self-efficacy Scale (GSE-10): It was designed in 1979 by Schwartzer and Jerusalem in order to assess general and social self-efficacy. It has 10 items all of which evaluate general self-efficacy. The construction of perceived self-efficacy indicates the individual’s optimistic view toward himself (Schwartzer, 1992). Individuals with a high level of self-efficacy believe that they can manage, solve the problems, and adapt themselves when they are faced with problems and difficult situations. Perceived self-efficacy facilitates determination of goals, energy devotion, hardiness against barriers, and recovery after failure. This scale is a self-report questionnaire used for adults (over 12 years old) in which the respondents are required to decide their agreement of disagreement using a 4-point Likert scale (from “Not correct al all” to “Completely correct”). To calculate the total score of the scale, the score of the 10 items need to be summed up. The score of this scale ranges from 1 to 40. In Germany, Schwartzer et al. (1992) calculated the internal consistency of this scale to be 0.84, 0.81 in Costa Rica and Spain, and 0.91 in China. Rajabi (2006) has calculated the reliability and validity coefficients of this scale and has reported it acceptable.

The lesson plan and intervention in the present study was a structured and group intervention that was composed of 10 weekly sessions held once a week for 1.5 hours (Antoni et al., 2007). In general, every session was composed of two sections; the first section dealt with skills of stress management and the second section included relaxation practices.

### 2.3 The Structure of the Sessions Was as Follow

First session: Administering the pre-test, explaining stress-causing factors and the importance of stress management, how to respond to stress-causing factors, creating a list of such factors, and relaxation practice

Second session: Getting aware of spontaneous thoughts, understanding the relationship between thoughts and feelings, understanding the physical symptoms, relaxation practice along with diaphragmatic breathing

Third session: Explaining the relationship between thoughts and excitements, identifying negative thoughts and understanding their effects on behavior, imagination and relaxation practice

Fourth session: Awareness of reasonable and unreasonable self-talks, relaxation practice in the form of imagination along with diaphragmatic breathing

Fifth session: Replacing reasonable thoughts, autogenetic training of heaviness and warmth feeling (sunlight meditation practice), relaxation practices in the form of mental imagination along with positive self-induction

Sixth session: Training efficient dealing, autogenic training of heartbeat, breath, stomach, and forehead

Seventh session: Administering responses of efficient dealing, autogenic training along with imagination and self-induction

Eighth session: Training anger management and mantra meditation

Ninth session: Training assertiveness, breath count meditation

Tenth session: Social support, a total review of the program, and creating a personal stress management plan.

The procedure of the intervention in each session was like this that the lessons of the last session were reviewed in the beginning of each session. Afterwards, lessons of that session were presented, and in the end the new materials were reviewed and educational homework was assigned.

## 3. Results

To describe the collected data, indices of central tendency and distribution were employed, whereby the mean and standard deviation were presented in the three phases of before the experiment, after the experiment, and during the follow-up, as indicated in Table 1 below.

**Table 1:** Mean and SD of the scores obtained in the pre-test, posttest, and follow-up

Variables	Experimental Group						Control Group					
	
	Pretest		Posttest		Follow-up		Pretest		Posttest		Follow-up	
	Mean	SD	Mean	SD	Mean	SD	Mean	SD	Mean	SD	Mean	SD
Anxiety	11.40	77.7	7.08	4.38	8.61	4.5	10.88	7.61	10.04	5.56	10.08	8.32
Hardiness	41.2	9.41	43.93	8.5	42.2	8.61	39.98	8.53	38.1	8.11	40.55	8.61
Self-efficacy	40.18	13.96	53.7	13.91	49.01	13.54	39.75	14.15	39.40	13.78	38.3	14.2

Table 1 indicates that the mean of anxiety for the experimental group before and after the study and during the follow-up was 11.40, 7.08, and 8.61, respectively. These figures were 10.88, 10.04, and 10.08 for the control group. The results indicate that the mean of anxiety in the experimental group dropped after the intervention and after the follow-up. However, there was no reduction of anxiety in the control group. Moreover, the mean of hardiness for the experimental group before and after the study and during the follow-up was 41.2, 43.93, and 42.2, respectively. These figures were 39.98, 38.1, and 40.55 for the control group. The results indicate that the mean of hardiness in the experimental group increased after the intervention and after the follow-up. However, there was no increase in hardiness in the control group. In addition, the mean of general self-efficacy for the experimental group before and after the study and during the follow-up was 40.18, 53.7, and 49.01, respectively. These figures were 39.75, 39.4, and 38.3 for the control group. The results indicate that the mean of general self-efficacy in the experimental group remarkably increased after the intervention and after the follow-up. However, there was no increase in general self-efficacy in the control group.

The degree of freedom for the groups was 4 and the error freedom degree was 25.

Table 2 indicates that MANOVA tests for the control and experiment groups were significant. These results show that there was a significant difference between the two groups in terms of anxiety, psychological hardiness, and general self-efficacy (p<0.001). In order to figure out the difference, the results are presented in Table 3 below.

**Table 2:** MABOVA test of the dependent variables in the experimental and control groups

	Test Criteria	Value	F ratio	P-value	η Square
Group	Pillai’s Trace	0.8	55.3	0.0001	0.46
	Wilks’s lambda	0.06	55.3	0.0001	0.68
	Hoteling Effect	16.5	55.3	0.0001	0.81
	The largest root of Ray	16.5	55.3	0.0001	0.89

**Table 3:** Levene’s test for equality of variances in the two groups based on the dependent variables (anxiety, psychological hardiness, and general self-efficacy)

Variables	F ratio	P-value
The level of anxiety		
The difference between the pretest and posttest	6.7	P<0.01
The difference between posttest and follow-up	6.6	P<0.01
The level of psychological hardiness		
The difference between the pretest and posttest	7.1	P<0.03
The difference between posttest and follow-up	7.6	P<0.01
The level of general self-efficacy		
The difference between the pretest and posttest	6.3	P<0.01
The difference between posttest and follow-up	5.5	P<0.01

Table 3 presents the results of Levene’s test for equality of variances of error in the two groups for the dependent variables (anxiety, psychological hardiness, and general self-efficacy). As indicated in Table 3, there was a significant difference between the two groups after the experiment and during the follow-up stages in terms of anxiety (p<0.01). Moreover, there was a significant difference between the two groups during these two stages in regard with their psychological hardiness (p<0.01). There was also a significant difference between the two groups regarding their general self-efficacy (p<0.01). According to Table 3, the proportion of the F ratios before and after the intervention and during the follow-up was significant in the levels of anxiety, hardiness, and self-efficacy.

According to the results of the present study, it can be concluded that cognitive-behavioral-therapy-based (CBT-based) stress management training has a favorable effect on a reduction in anxiety and an increase in psychological hardiness and general self-efficacy.

## 4. Discussion and Conclusion

The present study utilized a quasi-experimental method to assess the rate of effectiveness of stress group management training on anxiety, psychological hardiness, and general self-efficacy among university students. In so doing, a stress management educational package was used for the experimental group, and its effects on anxiety, psychological hardiness, and general self-efficacy was measured.

It was concluded that group training of stress management caused a reduction in anxiety among university students, such that their anxiety dropped significantly after the intervention. This finding is in agreement with those of the studies conducted by Timmerman et al. (1998), Halamandariz and Power (1999), Koegh et al. (2005), Soltani (2004), Kaviyani et al. (2007), and Shirbim et al. (2008). The results reported by these researchers indicated that stress management training can improve stress, tension, and social dysfunction.

The results of the present study also indicated that the students’ psychological hardiness increased remarkably after the educational intervention, i.e. group training of stress management resulted in an increase in psychological hardiness. This finding is in agreement with those of the studies conducted by Florian et al. (2012) and Brooks (2003). There are few studies in this field, and there is no study conducted on this issue in Iran, which limits the generalizability of the results. Therefore, it should be investigated in a larger sample in the future studies.

Moreover, the results of the present study indicated that group training of stress management can enhance general self-efficacy among university students. This finding is in line with those of the studies conducted by Hardin et al. (2002) and Abolghasemi et al. (2012). Life skill is one of the stress management and mental pressure skills, which strengthens the ability of uniqueness in the individual, such that he feels committed and responsible toward his life, and activities like discussion and debate, lesson taking, activity in small groups results in presentation of different responses in unpredicted situations, which is the same self-efficacy. Self-efficacy influences how individuals think, feel, and behave. The level of self-efficacy relies on the individual’s choice of assignments, commitment, attempt, and skill acquisition (Yari, 1997).

In general, it can be stated that group plan of CBT-based stress management is effective in reduction of anxiety among university students; it enhances psychological hardiness and self-efficacy among them.

One of the limitations of the present study was that the student’s homework was not controlled by the researchers. Moreover, personal and emotional variables were not controlled. Therefore, it is suggested that more reliable results can be achieved by increasing the education time and utilizing other therapeutic methods that can influence anxiety, psychological hardiness, and general self-efficacy.
